# A Novel De Novo Missense Variant in Netrin‐1 (*NTN1*) Associated With Chorioretinal Coloboma, Sensorineural Hearing Loss and Polydactyly

**DOI:** 10.1111/cge.14651

**Published:** 2024-12-08

**Authors:** Maria Toms, Cara Heppell, Nicholas Owen, Samantha Malka, Mariya Moosajee, Genomics England Research Consortium

**Affiliations:** ^1^ Development, Ageing and Disease UCL Institute of Ophthalmology London UK; ^2^ The Francis Crick Institute London UK; ^3^ Department of Genetics Moorfields Eye Hospital NHS Foundation Trust London UK

**Keywords:** coloboma, microphthalmia, netrin‐1, polydactyly, sensorineural hearing loss

## Abstract

Microphthalmia, anophthalmia and coloboma (MAC) comprise a highly heterogeneous spectrum of congenital ocular malformations with an estimated incidence of 1 in 5000 to 1 in 30 000 live births. Although there is likely to be a genetic component in the majority of cases, many remain without a molecular diagnosis. Netrin‐1 was previously identified as a mediator of optic fissure closure from transcriptome analyses of chick and zebrafish and was shown to cause ocular coloboma when knocked out in both mouse and zebrafish. Here, we report the first patient with chorioretinal coloboma and microphthalmia harbouring a novel heterozygous likely pathogenic *NTN1* missense variant, c.1483T>A p.(Tyr495Asn), validating a conserved gene function in ocular development. In addition, the patient displayed bilateral sensorineural hearing loss which was investigated by examining the sensory hair cells of *ntn1a* morphant zebrafish, suggesting a role for netrin‐1 in hair cell development.

## Graphical abstract

A novel heterozygous de novo *NTN1* missense variant was identified in a patient with chorioretinal coloboma, sensorineural deafness and polydactyly, through screening of micropthalmia anophthalmia coloboma (MAC) patients in the Genomics England 100 000 Genomes Project dataset. Gene knockdown of *ntn1a* in zebrafish caused defects in the eye and ear.

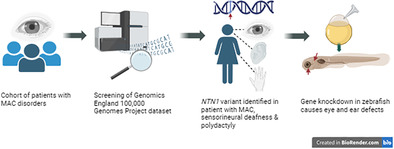

## 1 Introduction

Microphthalmia, anophthalmia and ocular coloboma (MAC) comprise a spectrum of congenital ocular malformations, ranging in severity from complete absence of an eye (anophthalmia) or a small (axial length ≥ 2 standard deviations [SD] below normal) under‐developed eye (microphthalmia), to incomplete fusion of the optic fissure (ocular coloboma) on the inferior aspect of the developing optic cup by 7 weeks gestation [1, 2]. Multiple ocular tissues may be affected by colobomatous defects, including the iris, ciliary body, retina, retinal pigment epithelium, choroid, optic disc and macula, with varying degrees of visual loss in affected patients. The prevalence of MAC has been estimated as 1 in 5000–30 000 live births and contributes to a significant proportion (up to 15%) of severe visual impairment or blindness in children globally [3, 4].

MAC is a genetically and clinically heterogeneous group of conditions. It can be present unilaterally or bilaterally with a combination of ocular malformations within or between eyes. MAC can be simplex, however, it is often associated with additional ocular (complex) and non‐ocular (syndromic) abnormalities. Although both genetic and environmental factors can be involved in MAC by impairing the development of the eye from week 3 of gestation, there is a genetic basis for the majority of cases and over 120 MAC‐associated genes have been identified to date [5]. These genes play diverse roles in the molecular signalling involved in the development of eyes and other organs and tissues, contributing to the clinically heterogeneous nature of MAC [2, 6]. Unilateral cases of MAC have typically shown lower rates of molecular diagnosis (~10%–20%), while a molecular diagnosis can be established in more than half of more severe or bilateral cases [7, 8, 9]; however, recent prospective studies have shown a 33% molecular diagnostic rate for MAC patients, with no difference between unilateral and bilateral cases [6]. Identifying the underlying genetic cause can inform appropriate disease management, genetic counselling and family planning, as well as aid in the development of effective therapeutic strategies.

*NTN1* has been highlighted as a candidate causative gene for MAC disorders from previous transcriptome analyses that identified the gene as a mediator of optic fissure closure in wild‐type chick [10] and zebrafish [11] embryos. *NTN1* encodes netrin‐1 (NTN1), a member of a family of laminin‐related secreted proteins that influence the development of several tissues by mediating cellular migration, adhesion and interactions [12]. Evidence of NTN1 function in optic fissure closure was provided by an ocular coloboma phenotype in zebrafish where *ntn1a* was knocked down [11] or knocked out [10], with the latter also showing microphthalmia. This has been further corroborated by optic fissure defects observed in *Ntn1* ^−/−^ mice [10].

Through screening of MAC patients in the Genomics England (GEL) 100 000 Genomes Project (GE100KGP) dataset, we have identified a novel heterozygous de novo *NTN1* missense mutation NM_004822.3:c.1483T>A p.(Tyr495Asn) in a patient presenting with unilateral left microcornea and chorioretinal coloboma, bilateral sensorineural hearing loss (SNHL) and right hand polydactyly. This individual was unsolved via the GEL analysis pathway, which included scrutiny of MAC and deafness gene panels. In addition to the ocular phenotype seen in animal disease models, we investigated the role of *NTN1* in sensory hair cell development and observed defects in the neuromast and inner ear hair cells of *ntn1a* morphant zebrafish larvae.

## 2 Materials and Methods

### 2.1 Patient Description

This study was approved by Moorfields Eye Hospital and the National Research Ethics Committee and was conducted in adherence to the tenets of the Declaration of Helsinki; informed written consent was obtained from the participant [13]. Ophthalmic evaluation included full orthoptic assessment, best corrected visual acuity using LogMAR, slit lamp examination, and fundus examination recorded with anterior segment and fundus colour imaging. Investigations included ultrasound biomicroscopy to measure axial length and anterior chamber depth, widefield optos colour and autofluorescence imaging.

### 2.2 Whole Genome Data Analysis

WGS analysis was performed through the Genomics England 100 000 Genomes Project (GE100KGP) [14]. Briefly, genomic DNA was processed and sequenced using the Illumina HiSeq X Ten sequencing platform (TruSeq DNA PCR Free Sample preparation, Illumina Inc.). Reads were aligned to build GRCh38 of the human genome using Isacc (Illumina Inc.) and variants (single‐nucleotide) and indels (insertion or deletions) identified using Platypus [15] (version 0.8.1; Wellcome Trust Centre for Human Genetics). From the undiagnosed proband cohort, rare variants predicted to alter the protein sequence were reviewed in those with suitable HPO terms present (microphthalmia HP:0000568, anophthalmia HP:0000528, true anophthalmia HP:0011478 or coloboma HP:0000589). Variants were annotated using Cellbase (https://github.com/opencb/cellbase) and variant filtering performed using minor allele frequency in publicly available data sets and predicted variant effect. Variants were prioritised using in house gene panels as well as the Structural Eye Disease virtual panel (version 3.0) from PanelApp [5] and analysis of de novo coding variants was undertaken in parent‐proband trios to identify candidate variants in novel genes. Variants were confirmed through interrogation of the paired‐end reads in Integrative Genomics Viewer (IGV) [16].

### 2.3 Zebrafish Husbandry and Microinjection

Adult zebrafish (wild‐type, AB strain) were maintained at the UCL main campus zebrafish facility under standard husbandry conditions and embryos were obtained by natural spawning [17]. Morpholino knockdown of *ntn1a* was performed as previously described [11]. Two independent microinjection experiments were performed to produce larvae for hair cell studies. Zebrafish were maintained according to institutional regulations for the care and use of laboratory animals under the UK Animals Scientific Procedures Act and the UCL Animal Welfare and Ethical Review Body (Licence no. PPL PC916FDE7). All approved standard protocols followed the guidelines of the ARVO Statement for the Use of Animals in Ophthalmic and Vision Research Ethics.

### 2.4 Wholemount Hair Cell Staining

Larval zebrafish at 5 days post fertilisation (dpf) were fixed with 4% PFA/PBS overnight at 4°C. Zebrafish were washed in PBS and incubated in PBS/1% Triton‐X for 4 days to dissolve the otoliths. The larvae were blocked in 20% normal goat serum in PBS‐1% Triton‐X for 1 h at room temperature before incubating with anti‐acetylated tubulin (Sigma‐Aldrich #T6793; 1:500) diluted in 1% normal goat serum in PBS‐0.5% Triton‐X overnight at 4°C. Washes with PBS‐1% Triton‐X was performed before incubating for 2 h at room temperature with anti‐mouse Alexa Fluor 488 secondary antibody (Thermo Fisher; 1:500) and Alexa Fluor 647 Phalloidin (Thermo Fisher #A22287; 1:10) diluted in 1% normal goat serum in PBS‐0.5% Triton‐X. The samples were washed with PBS‐1% Triton‐X before mounting in Prolong Gold Antifade Mountant (Thermo Fisher) on self‐made multi‐well microscope slides. Fluorescent hair cells were visualised using Zeiss LSM 700 AxioImage M.1 upright confocal microscope.

## 3 Results

### 3.1 Clinical Phenotyping

**FIGURE 1:**
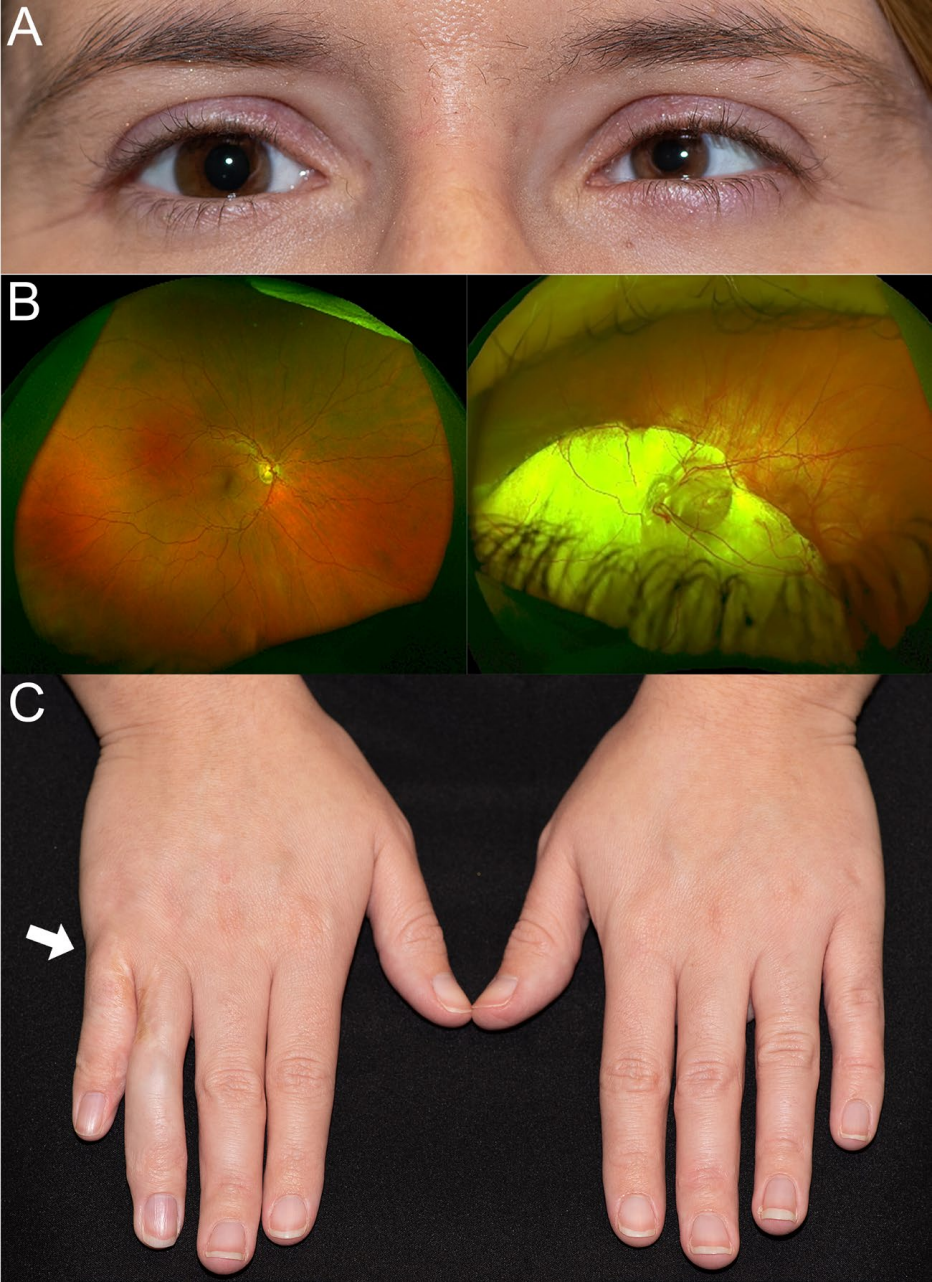
Clinical features of the patient with a heterozygous missense variant c.1483T>A p.(Tyr495Asn) in *NTN1*. (A) Facial photo showing unilateral left microphthalmia and microcornea. (B) Widefield colour fundus imaging showing extensive left chorioretinal coloboma involving the optic disc and macula, right fundus is normal. (C) Clinical photograph of both hands with white arrow pointing to right hand indicating site of extra digit (polydactyly), which was surgically removed from between ring and little finger.

The patient presented here was identified through processing a subset of the GE100KGP data. The patient was a 30‐year‐old White British female from a non‐consanguineous family. The ophthalmological features included unilateral left microcornea, left esotropia and left chorioretinal coloboma involving the optic disc and macula (Figure 1A,B). The right eye was healthy and unaffected. Best corrected visual acuity was 0.04 LogMAR in the right eye, and 1.40 LogMAR in the left eye. Axial length was 23.36 mm in the right eye and 26.81 mm in the left eye, anterior chamber depth was 3.63 mm in the right eye and 2.46 mm in the left eye, using ultrasound biomicroscopy. Corneal diameter measurements were 12 mm in the right eye and 8 mm in the left eye.

The patient also had bilateral symmetrical moderate‐to‐severe SNHL for which she wore hearing aids, mesoaxial hand polydactyly with an additional digit between the third and fourth fingers on the right hand, which had been excised surgically (Figure 1C), and absence of a flexion crease at the right distal interphalangeal (DIP) joint of the fourth finger, which she cannot flex. The neurological exam of the patient was normal. She had atrophic acneiform scarring on her back and one café au lait spot on her right lower back. She had a right inguinal hernia at age 12. The patient had no other affected family members.

### 3.2 Analysis of *NTN1* Variant

The identified proband harboured a heterozygous de novo variant in Netrin1 (*NTN1*) gene (NM_004822.3:c.1483T>A p.(Tyr495Asn)) absent in population databases [18]. Trio WGS analysis showed that the *NTN1* variant was present in the proband, but absent in both unaffected parents. The variant was classified on the basis of the American College of Medical Genetics and Genomics/Association of Molecular Pathology (ACMG/AMP) criteria as likely pathogenic (PP3 moderate; PM1 supporting; PM2 supporting; PS2 strong) [19]. The variant is located within the C‐terminal netrin‐like (NTR) domain coding region of *NTN1* and was predicted to be deleterious by Provean (−6.81), Polyphen‐2 (0.986), SIFT (0.002), MutationTaster (1) and AlphaMissense (0.806). This variant is 4 nucleotides from the end of exon 6 of *NTN1* and may affect splicing (MutationTaster score of 0.93 for splice donor loss). *NTN1* is highly constrained against loss of function variants (pLI = 1). This amino acid is conserved across vertebrate genomes, with a phyloP100 conservation score of 7.547.

To date, no complete structural information of human *NTN1* was available, however, the NTR domain (Netrin module) consists of protein domains found in the C‐terminal of netrins, complement proteins, type I procollagen C‐proteinase enhancer proteins (PCOLCEs), as well as in the N‐terminal domains of tissue inhibitors of metalloproteinases (TIMPs) [12, 20]. The NTR of *NTN1* encompasses amino acids 472 to 601 and contains six conserved cysteines, using PhyreRisk the modelled protein structure (PDB 1UAP_A) consists of 125 amino acids with 100% identity to the C‐terminal region of *NTN1* (residues 470–595) [12, 20]. No other pathogenic genotypes or Tier 1 variants were detected in genes known to cause deafness or MAC in this patient [5]. The following Tier 2 variant was found in a gene not included on the MAC panel, but deemed not likely to be contributing to the patient's phenotype: *RUNX2* NM_001024630.4:c.820C>A; p.(Pro274Thr), a heterozygous missense variant inherited from the patient's unaffected mother. It was classified as likely benign, with evidence as follows: BS2 (strong); BP4 (moderate); PP2 (supporting).

### 3.3 Hair Cell Analysis in *ntn1a* Morphant Zebrafish Larvae

**FIGURE 2:**
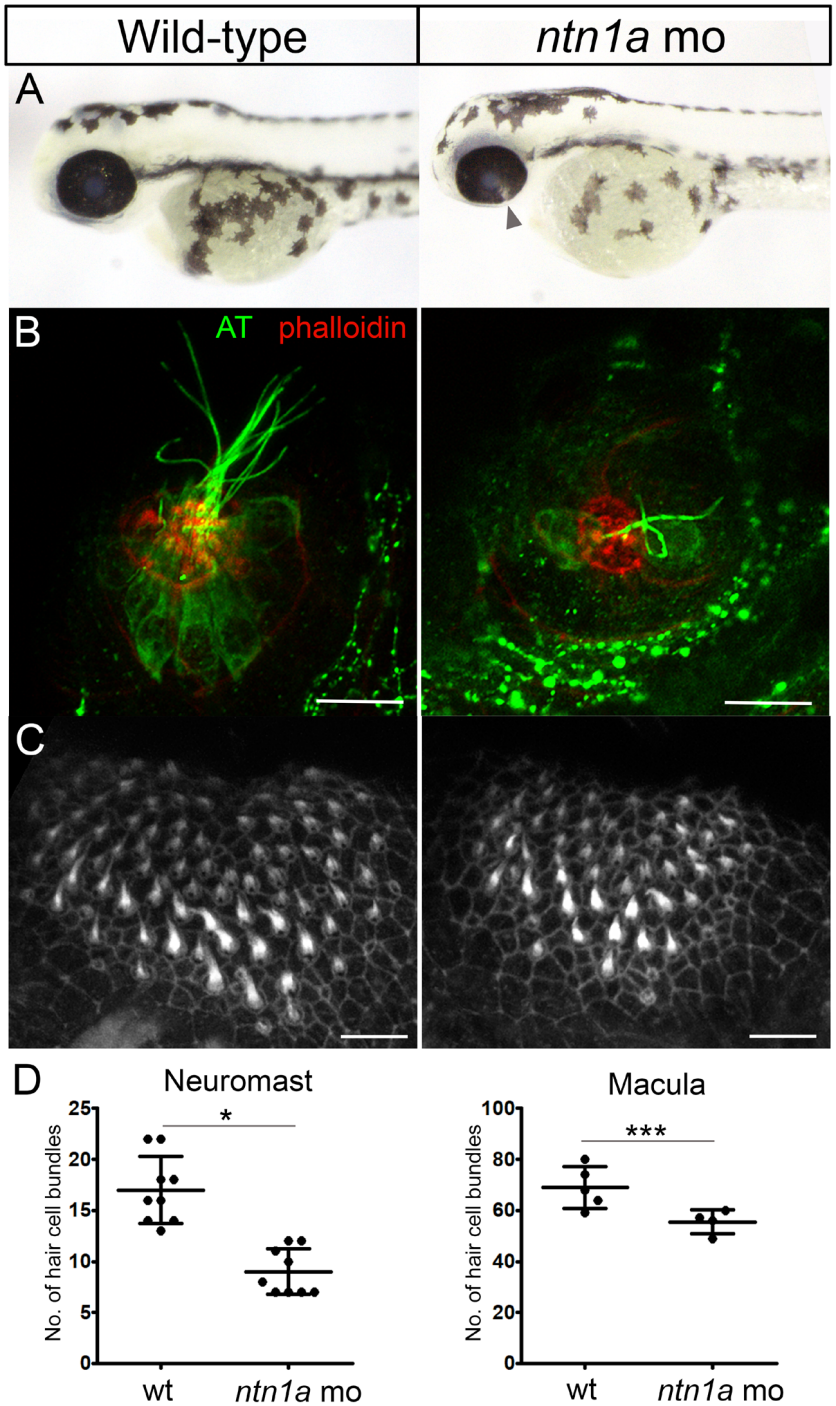
Ocular coloboma and sensory hair cell defects in *ntn1a* morphant zebrafish. (A) Fusion of the optic fissure was not complete in *ntn1a* morphant embryos at 56 h post‐fertilisation. The arrow indicates the ocular coloboma. (B) The lateral line neuromasts were visualised in control and *ntn1a* larvae at 5 days post‐fertilisation (dpf). Anti‐acetylated tubulin (AT, green) detected the hair cell bodies and kinocilia and Alexa Fluor 647 Phalloidin (red) stained the stereociliary hair bundles. The *ntn1a* neuromasts lacked hair cell bodies, hair bundles and kinocilia. (C) The stereociliary hair bundles of the anterior macula in the inner ear were visualised used Alexa Fluor 647 Phalloidin (white). The hair bundle morphology appeared to be normal in *ntn1a* at 5 dpf, but the hair bundles were reduced in number. (D) The bar charts show that the numbers of hair bundles per neuromast (*n* = 9, *p < 0.05) and per anterior macula (*n* = 5, ***p < 0.001) were significantly reduced in the *ntn1a* morphant compared with control larvae at 5 dpf. Scale bars = 10 μm.

*ntn1a* has been identified previously as the zebrafish orthologue of the human *NTN1* gene, with 86% similarity in an amino acid alignment [21]. Zebrafish also possess a paralogue *ntn1b*, however, transcriptomic analysis did not reveal this gene to be involved in zebrafish optic fissure fusion [11]. Knockdown of *ntn1a* in zebrafish using a translation‐blocking antisense morpholino resulted in optic fissure closure defects apparent in all injected embryos by 56 h post‐fertilisation (Figure 2A). To assess the potential involvement of *NTN1* in sensory hair cell development, the hair cells of the inner ear and lateral line were visualised in *ntn1a* morphant zebrafish at 5 dpf. The hair cells of the lateral line neuromasts were found to be defective in the morphant larvae, lacking hair cell bodies, stereociliary hair bundles and kinocilia (Figure 2B). There was a significant reduction in the number of hair bundles per neuromast, with a mean of 9 ± 2.2 compared with 17 ± 3.3 in the wild‐type controls (*p* < 0.001, *n* = 9) (Figure 2D). The hair cell bundles of the inner ear (anterior and posterior maculae) appeared to have a normal morphology in the morphant larvae at 5 dpf (Figure 2C). However, it was found that the number of macular hair bundles were reduced in number, with a mean of 55.5 ± 4.7 per anterior macula in the *ntn1a* morphants compared with 69 ± 8.2 in the control larvae (*p* < 0.05, *n* = 5) (Figure 2D).

## 4 Discussion

*NTN1* is a laminin‐related secreted protein gene previously identified as a mediator of optic fissure fusion through multi‐species transcriptomic analysis and animal knockout studies [10, 11]. In the present study, we have identified the first reported patient with ocular maldevelopment on the MAC spectrum harbouring a novel heterozygous *NTN1* variant. Furthermore, gene knockdown in zebrafish indicated that the bilateral SNHL displayed by the same patient may also be a result of defective *NTN1*. To the best of our knowledge, this is the first MAC‐associated *NTN1* variant identified in humans.

Netrins are involved in cellular migration, adhesion and cell–cell interactions. They have a common structure with a laminin N‐terminal, three cysteine rich EGF domains and C‐terminal NTR domain [22]. They mediate axonal migration through the binding of the laminin N‐terminal and EGF domains to the transmembrane receptors Deleted in Colorectal Cancer (DCC) and UNC5 [12, 22]. The function of the NTR is not known [23], however, its involvement in nucleolar localisation and control of metalloproteinases has been theorised [20, 24]. The NTR has also been shown to have an adhesive function in embryonic pancreatic epithelial cells, and mammary gland development and lung branching morphogenesis in mice models [25, 26, 27]. In the present study, the variant described is predicted to be a damaging variant by multiple variant effect prediction software tools, and may affect splicing, potentially causing significant changes to the C‐terminal protein sequence. This variant may impair the adhesive properties of NTN1 in the optic fissure, preventing fusion and resulting in the chorioretinal coloboma observed in this patient.

Optic fissure closure involves the fusion of the ventral optic fissure [28]. During embryogenesis, neuroepithelium evaginates from the diencephalon forming the optic vesicle. Following contact with the surface ectoderm, the optic vesicle proceeds to invaginate forming the optic cup, consisting of an inner neural retinal layer and outer retinal pigment epithelial layer. Optic fissure closure begins at week five and involves the fusion of epithelium at the optic fissure margins. This process is essential for ensuring circumferential continuity of the globe with failure resulting in ocular colobomas [28]. High expression of *NTN1* at the optic fissure has been seen in mice, zebrafish, chick and human foetal tissue, supporting a role in optic fissure fusion [10, 11]. Defects in optic fissure closure in chick, zebrafish and mouse *NTN1* knockout models further corroborate this function in mediating tissue fusion, however, the mechanism by which it achieves this is not known [10, 11].

NTN1 acts in various signalling pathways and biological processes across a range of organs and tissues, including axon guidance and vascular morphogenesis, and therefore has been implicated in a range of pathologies in both patients and animal models. NTN1 is a bifunctional cue that mediates axonal attraction and repulsion. In association with DCC, it leads to axonal attraction or repulsion and, with UNC5, axonal repulsion, seen in Drosophila, chick and mice models [12, 29, 30]. These axonal cues influence neuronal and blood vessel migration in mice and zebrafish models [31, 32]. In the development of the nervous system, NTN1 acts as a midline chemoattractant and in ocular development, it has been reported to mediate guidance of retinal ganglion cell axons locally at the optic disc in mouse models [33, 34]. *Ntn1*, along with *Dcc* and *Unc5* homologues, has been found to be expressed in the developing limb bud during mouse development [35], supporting a role for *NTN1* in the hand anomalies reported in our patient. Furthermore, distal arthrogryposis affecting the fingers and toes was reported among several abnormalities in a patient harbouring a 47, XYY karyotype and two rare de novo CNVs, which included a 12‐kb deletion between the second and third exons of *NTN1* [36].

Despite its widespread functions, there have been limited reports of disease‐causing variants identified in *NTN1*. Previously, Méneret et al. reported heterozygous *NTN1* variants in three patients with congenital mirror movements (CMM; OMIM #618264), a disorder characterised by involuntary movements of one hand that mirror intentional movements of the opposite hand [37]. The anatomy of the corticospinal tract was found to be abnormal in the patients. The identified variants comprised two missense variants and one in‐frame deletion in the NTR of *NTN1*. It is unclear why there was an absence of other manifestations in the patients with CMM, and why the *NTN1* variant reported in our patient caused abnormalities distinct from those with CMM. Variants in many MAC‐associated genes, such as *PAX6*, typically cause conditions with high levels of phenotypic variability, often attributed to the diverse functions of these genes in multiple tissues, organs and signalling pathways, as well as the presence of genetic modifiers and environmental factors. Méneret et al. hypothesized that the CMM‐*NTN1* variants caused structural changes, which cause protein instability or prevent secretion, leading to reduced NTN1 in the extracellular space. It is unknown whether the missense variant reported here affects secretion, but it is predicted to affect splicing of NTN1 by disrupting a splice donor site; this could potentially alter the amino acid sequence of the protein downstream of the variant, disturbing essential interactions with other proteins. Further experimental work is required to investigate how the p.(Tyr495Asn) variant affects protein localisation and function.

*NTN1* variants have also been associated with cleft lip in patients [21, 38], in addition to non‐syndromic heart defects, cleft palate and semicircular canal defects in mice and zebrafish models, consistent with NTN1 as a mediator of tissue fusion [21, 36, 39]. Fusion defects are also seen in patients with CHARGE syndrome, one of the syndromic associations of MAC. It is characterised by colobomas, heart defects, choanal atresia, growth retardation and genital and ear anomalies, and associated fusion defects include colobomas, heart defects, cleft palate and delayed fusion of the semicircular canals [40]. It is associated with *CHD7* variants in the majority of patients [41]. Delayed *Ntn1* expression has been reported in mice with loss of *Chd7* resulting in delayed fusion of the semicircular canals [39].

The defective hair cells of the lateral line neuromasts identified in *ntn1a* knockdown zebrafish suggests the bilateral SNHL present in the patient may also be attributable to the variant in *NTN1*. This supports prior literature reporting the key role played by *Ntn1* in mediating protective effects on cochlear hair cells in mice, thereby preventing SNHL [42]. Insulin growth factor 1 (IGF‐1) is released in response to damage to hair cells, which, on binding to its receptor, activates the phosphatidylinositol 3‐kinase (PI3K)/Akt and mitogen‐activated protein kinases (MAPK)/extracellular signal‐regulated kinases (ERK) pathways [43, 44]. The downstream effects of these pathways lead to the upregulation of NTN1, which binds to UNC5B and inhibits apoptosis of hair cells [42]. However, although reduced in number, the hair cell bundle morphology in the inner ear of morphant larvae was unaffected. Defective development of the semicircular canals, for which *Ntn1* is required [45], could also explain the SNHL seen in this patient.

In conclusion, we report a novel *NTN1* variant likely causing chorioretinal coloboma, SNHL and polydactyly in our patient. This finding further substantiates previous evidence of the role played by *NTN1* in optic fissure closure. The identification of another causative gene for MAC will enhance diagnostic capacity, facilitating the management of patients with genetic counselling and family planning and presents a potential target for novel therapeutic interventions. Further investigation into the mechanism by which this variant results in the MAC and SNHL phenotype is warranted.

## Author Contributions

Conceptualization: M.M. Formal analysis: N.O., M.T., C.H, and S.M. Funding acquisition: M.M. Investigation: N.O. and M.T. Visualization: M.T. and M.M. Supervision: M.M. Writing – original draft: M.T., C.H. and N.O. Writing – review and editing: M.T., M.M. and S.M.

## Conflicts of Interest

The authors declare no conflicts of interest.

## Data Availability

The genome data are available in the secure Genomics England Research Environment. Clinical data and imaging are stored within the respective NHS trust's electronic patient records.
